# Pharmacological Benefits of Triphala: A Perspective for Allergic Rhinitis

**DOI:** 10.3389/fphar.2021.628198

**Published:** 2021-04-30

**Authors:** Salinee Jantrapirom, Pannaphak Hirunsatitpron, Saranyapin Potikanond, Wutigri Nimlamool, Nutthiya Hanprasertpong

**Affiliations:** ^1^ Department of Pharmacology, Faculty of Medicine, Chiang Mai University, Chiang Mai, Thailand; ^2^ Drosophila Center for Human Diseases and Drug Discovery (DHD), Faculty of Medicine, Chiang Mai, University, Chiang Mai, Thailand; ^3^ Graduate School, Chiang Mai University, Chiang Mai, Thailand

**Keywords:** allergic rhinitis, triphala, antioxidant effect, anti-inflamamtion, immunomodulaion

## Abstract

Allergic rhinitis (AR) is considered a major nasal condition impacting a large number of people around the world, and it is now becoming a global health problem. Because the underlying mechanisms of AR are complex, the development of single-drug treatment might not be enough to treat a wide spectrum of the disease. Although the standard guidelines classify and provide suitable diagnosis and treatment, the vast majority of people with AR are still without any means of controlling it. Moreover, the benefits of AR drugs are sometimes accompanied by undesirable side effects. Thus, it is becoming a significant challenge to find effective therapies with limited undesirable side effects for a majority of patients suffering from uncontrolled AR. Aller-7/NR-A2, a polyherbal formulation, has revealed promising results in patients by reducing nasal symptoms and eosinophil counts without serious adverse effects. Interestingly, three out of seven of the herbals in the Aller-7/NR-A2 formulation are also found in an Ayurvedic polyherbal formulation known as “Triphala,” which is a potential candidate for the treatment of AR. However, there are no current studies that have examined the effects of Triphala on the disease. This review aims to describe the complexity of AR pathophysiology, currently available treatments, and the effects of Triphala on AR in order to help develop it as a promising alternative treatment in the future.

## Introduction

Although allergic rhinitis (AR), commonly known as hay fever, is not considered a serious condition or one with the potential for mortality, it might become a clinical burden if it were to become uncontrollable in a majority of patients. Epidemiological studies have revealed that patients with AR have a high probability of subsequently developing asthma, and a large proportion of asthmatic patients also have AR as a concomitant condition (Simons, 1999; Thomas, 2006; Vujnovic and Domuz, 2018). Moreover, prolonged AR symptoms, particularly nasal congestion, can lead to the development of obstructive sleep apnea (Young et al., 1997). Currently, three acceptable strategies for managing AR are employed: 1) avoiding sensitized allergenic exposure, 2) starting pharmacotherapy, and 3) starting immunotherapy (Klimek et al., 2019). However, to achieve final outcomes, it is often suggested to patients that they use long-term multiple therapies, which sometimes come with problems of compliance, leading to failures in treatment (Small et al., 2018). Therefore, combinations of two drugs in one device, such as antihistamine/steroid or anticholinergic/steroid, are becoming novel strategic treatments that have been found to be more effective than using a monotherapy (Seresirikachorn et al., 2018).

Several studies have highlighted the beneficial effects of phytochemicals in attenuating allergic responses and the overall symptoms of AR. Curcumin, ginger, ginseng, resveratrol, and quercetin compounds have all shown effective results in relieving nasal symptoms and suppressing AR-related mediators in animal models by controlling the main anti-inflammatory mitogen-activated protein kinase/nuclear factor κB (MAPK/NF-κB) pathway (Jung et al., 2011, 2013; Zhang et al., 2015; Kashiwabara et al., 2016; Kawamoto et al., 2016; Lv et al., 2018). A major component of ginger, 6-gingerol, possesses the ability to suppress T helper 2 (Th2) cytokines by attenuating the activation of MAPK/NF-κB signaling in ovalbumin (OVA)-sensitized spleen cells (Kawamoto et al., 2016). In addition, resveratrol nasal spray has recently been developed to overcome the problem of drug absorption, and specifically to retain bioactive activities in the affected area, such as the nasal mucosa. An intranasal resveratrol formulation has been shown to be successful in alleviating allergic symptoms and reducing eosinophils and mast cell infiltration in the nasal mucosa of both animals and AR patients (Lv et al., 2018). Moreover, children with AR have significantly reduced nasal symptoms following administration of a combined resveratrol and carboxymethyl β-glucan nasal spray (Miraglia Del Giudice et al., 2014).

Triphala possesses several activities that could be beneficial for respiratory and immune systems. However, there is no study directly evidencing the potential benefits of Triphala on AR, even though its constituent phytochemicals have been suggested to serve many aspects of AR therapeutic strategies. Therefore, it is important to review and clarify the possible mechanism of action of Triphala and its active constituents on AR. This review focuses mainly on the antioxidative, anti-inflammatory, immunomodulatory, and other effects of Triphala on the AR-affected respiratory system. The quality of the articles relevant to the scope of AR has been evaluated according to the guideline reported by Heinrich et al. (2020) and the Good Research for Comparative Effectiveness (GRACE) tool (Dreyer et al., 2016) (Supplementary Table S1).

## Allergic Rhinitis

AR is a condition of nasal hypersensitivity caused by an immunologically mediated inflammation by non-infectious stimulants or environmental allergens, such as pollen, dust, fungi, cockroaches, etc. AR is becoming a global health problem affecting approximately 10–40% of the global population (Björkstén et al., 2008; World Allergy Organization, 2013; Bernstein et al., 2016). Although it is not a condition that is associated with high morbidity and mortality, it generates a heavy burden and has a negative impact on the patients’ quality of life (QoL) by reducing performance at work and disturbing sleep. In some cases, the condition can persist throughout the patient’s life. The common symptoms of AR include nasal congestion, rhinorrhea, nasal itchiness and/or itchy eyes, sneezing, and postnasal drip (Klimek et al., 2019). Uncontrolled moderate or severe AR may also impact the control of asthma (Thomas, 2006).

**FIGURE 1:**
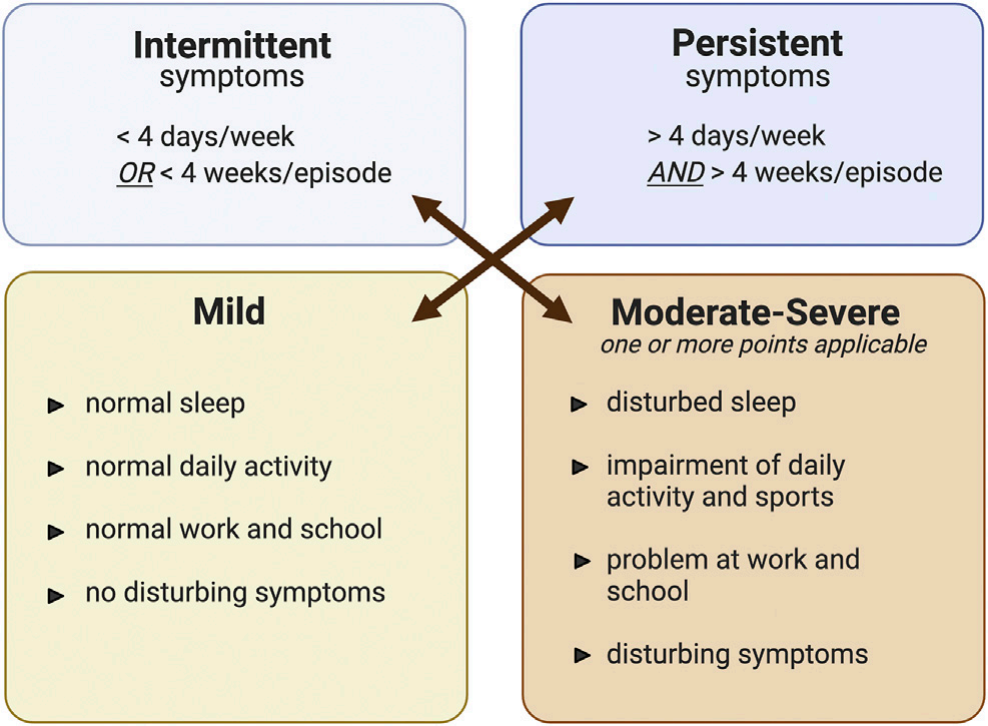
The classification of allergic rhinitis modified from ARIA guideline (Klimek et al., 2019). Classification of allergic rhinitis by the level of severity (mild or moderate-severe) and episode of symptom occurrence (intermittent or persistent). The figure was created by BioRender.com.

Currently, AR is classified according to the duration (intermittent or persistent) and severity (mild, moderate/severe) of symptoms. Intermittent AR is diagnosed if the frequency of symptoms is fewer than four times per week or a month per year. A frequency greater than this is classified as persistent. Moreover, the impact of the disease on a patient’s QoL is also used to evaluate the severity of the symptoms. Symptoms are considered to be mild if normal daily activities, such as sleep, study, and work, can be undertaken without troublesome or interfering symptoms. On the other hand, if a patient presents at least one symptomatic difficulty, then the symptoms are defined as moderate/severe (Figure 1) (Klimek et al., 2019). Therefore, consideration of both duration and severity is an important guide to choosing the most suitable treatment for each patient.

The key elements for AR management are as follows: 1) avoidance of suspected allergens in sensitized individuals, 2) providing appropriate therapeutic agents to reduce nasal symptoms and improve patients’ QoL, and/or 3) considering allergen-specific immunotherapy (AIT) (Varshney and Varshney, 2015). The list of medications used for AR, their mechanism of action, and their known adverse effects are listed in Table 1 (Bousquet et al., 2008; Klimek et al., 2019; Hossenbaccus et al., 2020). Although these treatments alleviate many AR-causing symptoms effectively, their use may also cause additional adverse effects.

**TABLE 1:** The medications for allergic rhinitis.

Treatment	Mechanism of action	Adverse effects
Antihistamines (oral)	Block H_1_ receptor on effector cells to reduce histamine-mediated symptoms such as rhinorrhea, sneezing, nasal itching, and eye symptoms	1st generation: Sedation, dry mouth, decrease cognitive function, orthostatic hypotention etc.
		2nd generation: Bitter taste (azelastine), rare cardiac arrhythmia in some drugs etc.
Antihistamines (intranasal, intraocular)	Block H_1_ receptor on effector cells located in nasal and ocular area to reduce histaminic effects as mentioned above	Nasal irritation and epistaxis, septal perforation, bitter taste (azelastine) etc.
Intranasal corticosteroids	Bind to glucocorticoid receptor leading to nasal inflammatory cells flux reduction	Nasal irritation, nasal dryness, epistaxis etc.
Oral/IM corticosteroids	Bind to glucocorticoid receptor leading to systematic inflammatory cells flux reduction	Adrenocortical insufficiency, infection, peptic ulcer, glaucoma etc.
		Depot injections may cause local tissue atrophy
Oral decongestants (sympathomimetic decongestants)	Stimulate systematic α adrenergic receptor to constrict dilated blood vessels	Hypertension, palpitations, restlessness, agitation, tremor, insomnia, headache, dry mucous membrane, urinary retention
Intranasal decongestants (sympathomimetic decongestants)	Stimulate nasal α adrenergic receptor to constrict dilated blood vessels located in nasal mucosa	Nasal irritation, nasal dryness, hypertension in some cases etc.
		Long term used may cause rebound congestion
Intranasal anticholinergics	Block muscarinic receptor leading to reduction of nasal mucosa gland activation	Nasal irritation, nasal dryness, epistaxis etc.
Leukotriene receptor antagonists (oral)	Block cysteinyl leukotriene receptor on effector cells to reduce vascular permeability and inflammatory cell recruitment	Urticaria, diarrhea, abdominal pain, agitation, suicidal thinking, hallucination etc.
Immunotherapies (subcutaneous, SCIT) (sublingual, SLIT)	Induce specific allergen tolerance and help restoration of immune system	Scit: Local irritation, swelling at the site of injection, anaphylaxis etc.
		Slit: Lip, mouth, and tongue irritation etc.

**FIGURE 2:**
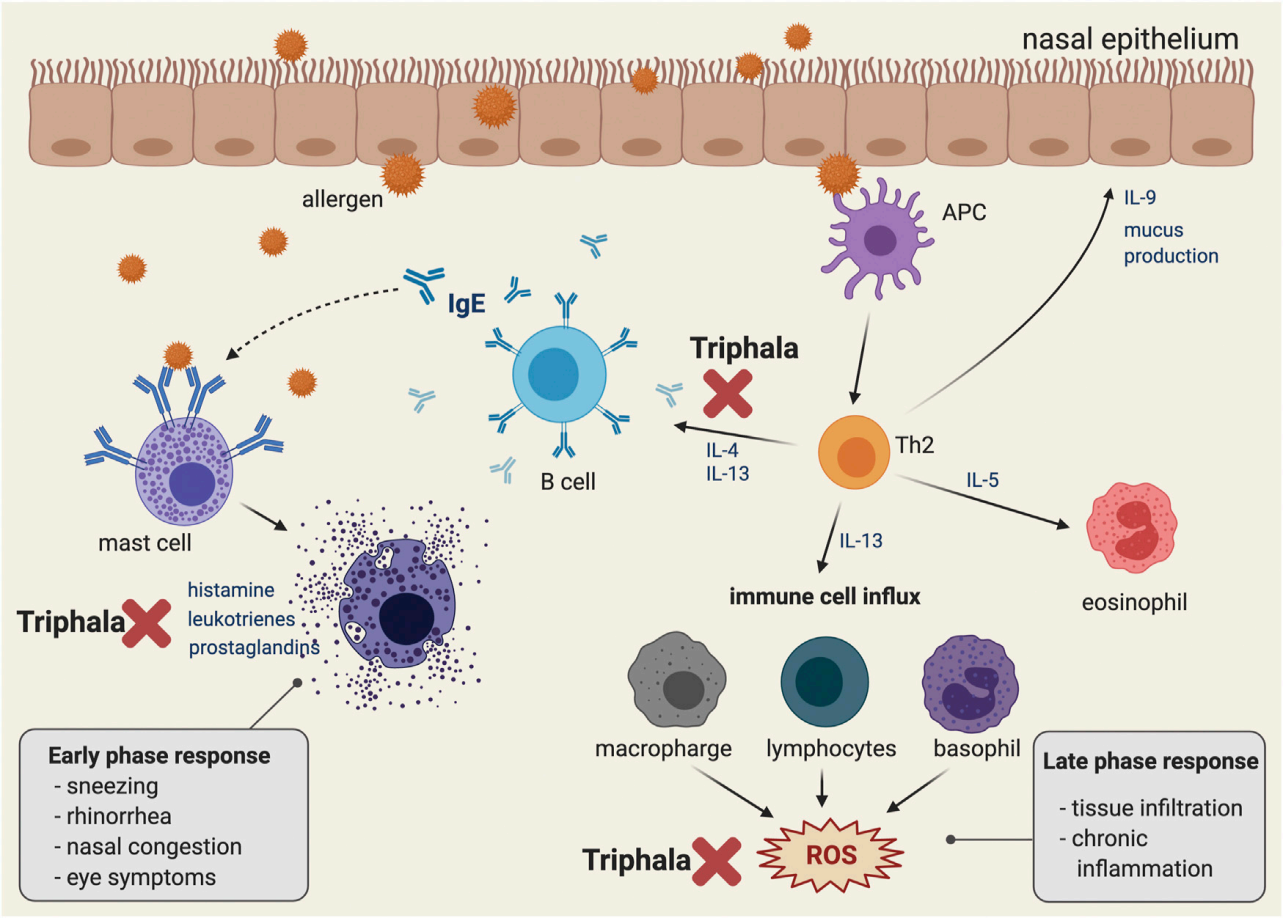
Pathophysiology of allergic rhinitis and the conceptual representative of a possible mechanism of action of Triphala in suppressing IgE dependent allergic reaction. A schematic illustration representing the pathophysiology of the IgE-associated allergic reaction. Immediately following exposure to the allergen, the latter is bound to the IgE on the surface of the mast cell and eventually activates mast cell degranulation, which releases inflammatory mediators. In addition, late phase and chronic allergic inflammation occur as a result of eosinophil, macrophage, lymphocyte, and basophil activation. Eventually, following these cell activations, the intracellular ROS are generated. Triphala may play an inhibitory role at the early phase response by suppressing the release of autacoids (including histamine, leukotrienes, and prostaglandins) and by down-regulating the production and secretion of IL-4 and IL-13, which are key AR mediators for enhancing inflammation and IgE production in B cells. Moreover, Triphala may help modulate the late phase response by scavenging the oxidative stress molecules and products. The figure was created by BioRender.com.

The pathophysiology of AR is quite complex (Figure 2), beginning with sensitization and early and late-phase responses (Min, 2010; Sin and Togias, 2011). Allergens can enter the submucosal area guided by antigen-presenting cells (APCs), which are mainly dendritic cells and macrophages, or pass through disturbed epithelial cells (Bharadwaj et al., 2007; Lambrecht and Hammad, 2010). Some allergens may have an additional protease-related activity to help them cleave the tight junctions between epithelial cells (Reithofer and Jahn-Schmid, 2017). After recognizing the antigen, the activated APCs then undergo maturation, migrate to regional lymph nodes, and present allergen peptides to naïve T cells (Th0). The presentation of peptides requires the involvement of major histocompatibility complex (MHC) class II molecules expressed on the APC surface, together with T cell receptors located on naïve T lymphocyte (Th0) surfaces (Bharadwaj et al., 2007). Depending on the cytokine-stimulated pattern, the Th0 cells can be differentiated into T helper type 1 (Th1) and T helper type 2 (Th2) cells (Zhu et al., 2010). Recognition of the antigenic peptides, together with activation by interleukin-4 (IL-4) derived from various resident cells, drives the Th0 cells to preferentially acquire the differentiated form of Th2 cells (Zhu et al., 2010). Furthermore, the release of IL-4, IL-5, and IL-13 by Th2 cells induces B-cell immunoglobulin class-switch recombination (Poulsen and Hummelshoj, 2007). The gene segments responsible for encoding the immunoglobulin heavy chain are rearranged in order to produce allergen-specific IgE antibodies (Gadermaier et al., 2014).

The early phase occurs in sensitized individuals within minutes of exposure to allergens. Soluble IgE antibodies, which are produced by B cells in the sensitization phase, locally diffuse and systematically enter the body fluids via lymphatic vessels (Burton and Oettgen, 2011). IgE can strongly bind to the tetrameric (αβγ2) high-affinity surface receptors for IgE (FcεRI) on tissue mast cells and basophils (Turner and Kinet, 1999). Any cells with surface-bound IgE antibodies are called sensitized cells, and they are prompted to respond to specific antigens through the membrane-bound IgE antibodies. The crosslinking of adjacent IgE antibodies by allergens induces aggregation of FcεRI and then triggers a rapid degranulation and secretion of various mediators, including histamine and tryptase (Turner and Kinet, 1999). Mast cells also release newly synthesized lipid mediators, including leukotrienes (LTC4, LTD4, and LTE4) and prostaglandins (PGD2) to help sustain inflammation by neutrophil and eosinophil infiltration (Fanning and Boyce, 2013).

There are many signs and symptoms associated with the early phase of AR, such as nasal pruritus, sneezing, and coughing caused by the histamine-stimulated sensory nerve endings of the trigeminal nerves (Taylor-Clark et al., 2005; Rosa and Fantozzi, 2013). The activation of sympathetics and parasymphathetics by histamines can also stimulate the mucous glands to produce a watery discharge that is presented as rhinorrhea (Al Suleimani and Walker, 2007). Histamines, together with leukotrienes and prostaglandins, act on blood vessels, causing vasodilation and nasal congestion (White, 1990).

The late phase response is characterized by recruitment of T cells, eosinophils, basophils, and neutrophils following a release of their various mediators, which leads to a continuation of the symptoms and inflammatory induction. This phase usually begins around 4–5 h following exposure to allergens and can last for 24 h. Some cytokines, such as IL-4, IL-13, and tumor necrosis factor-α (TNF-α) released from mast cells, have the ability to upregulate two adhesion molecules, which are vascular cell adhesion molecule-1 (VCAM-1) and intracellular cell adhesion molecule-1 (ICAM-1) (Lee et al., 1994). These adhesion molecules subsequently induce migration of basophils, eosinophils, neutrophils, and T cells to the nasal mucosa. Moreover, an activated form of group 2 innate lymphoid cells (ILC2), which are induced by mast cell-related cytokines (leukotrienes, prostaglandins, and platelet-activating factor (PAF)) play a role in continuing the inflammation by releasing large amounts of Th2 cytokines (Li and Hendriks, 2013). Neutrophils that are recruited into the nasal mucosa can harm the epithelium by inducing reactive oxygen species (ROS) and enzyme (protease, matrix metallopeptidase 9 (MMP-9), and myeloperoxidase) production (Lacy, 2006; Ventura et al., 2014). Persistent inflammation in the late phase is associated with tissue remodeling, which might also lead to alteration of organ function and nasal hyperresponsiveness (Galli et al., 2008).

## Triphala

Triphala, one of the polyherbal formulations used in Ayurvedic medicine, comprises the dried fruits of three plants, namely, *Terminalia chebula* Retz (Family Combretaceae R. Br), *Terminalia bellirica* (Gaertn.) Roxb (Family Combretaceae R. Br), and *Phyllanthus emblica* L (Family Phyllanthaceae Martinov). The composition of Triphala is derived from a rational combination of *T. chebula* Retz., *T. bellirica* (Gaertn.) Roxb., and *P. emblica* L., which are combined to balance the three pillars of life or elemental substances, known in Sanskrit as the three doshas, namely, Vata (elements of space and air), Kappha (elements of earth and water), and Pitta (elements of fire and water) (Peterson et al., 2017). Normally, the herbs are mixed in equal proportions (1:1:1), but sometimes the ratios can be modified to be one part *T. chebula* Retz., two parts *T. bellirica* (Gaertn.) Roxb., and three parts *P. emblica* L (1:2:3) (Gajendra et al., 2016) or one part each of *T. chebula* Retz. and *T. bellirica* (Gaertn.) Roxb. and two parts *P. emblica* L (1:1:2) (Ginsburg et al., 2009). According to a traditional Thai medicine textbook, Triphala is used to provide relief for different disease conditions related to the imbalance of water element (Kappha); specifically, the difficulty of breathing caused by excessive water-soluble element and mucus retention in the lungs. Although there is no specific definition of AR in Traditional Thai medicine, Triphala is used to reduce runny nose, cough, the viscosity of phlegm, sore and dry throat, and thirst. These symptoms are somehow related to AR symptoms.

Several phytochemicals have been detected in the fruits of these three plants. These include phenolic compounds (gallic acid, ellagic acid, chebulinic acid, chebulagic acid, and emblicanin A and B), flavonoids (quercetin and kaempferol), alkaloids (phyllantidine and phyllantin), ascorbic acid, carbohydrates, proteins, etc. (Parveen et al., 2018). The amount of each phytochemical varies depending on the methods used to extract it, the solvents used, and the region in which the parent plant was grown (Parveen et al., 2018). One study by Liu et al. found that samples collected from different regions in China contained a range of phenolic contents in *P. emblica* L. methanolic extracts (Liu et al., 2008). Moreover, methanolic extraction appeared to yield more phytochemicals than aqueous extraction (Parveen et al., 2018). Therefore, characterization of the compound to be extracted should be a mandatory procedure before undertaking extraction experiments.

The phytochemicals contained in Triphala may be beneficial for the treatment of AR. Gallic acid and quercetin are prominent phenolic compounds and are well-known antioxidants in AR-related studies. Gallic acid was shown to alleviate nasal inflammation by shifting the immune response toward Th1 in a mouse model of OVA-induced AR (Fan et al., 2019). The increment of Th2 cytokines, including IL-4, IL-5, IL-13, and IL-17 was attenuated, whereas Th1-related cytokines, including interferon-γ (IFN-γ) and IL-12, were upregulated in nasal larval fluid (NALF) upon treatment with gallic acid (Fan et al., 2019). Moreover, histopathological improvements such as nasal mucosa thickening, goblet cell hyperplasia, and eosinophil infiltration, have been observed in a gallic acid-treated group of patients (Fan et al., 2019). One study also found that the AR-positive effects of gallic acid were caused by inhibition of pro-inflammatory cytokines and histamine release via the modulation of cyclic adenosine monophosphate (cAMP), intracellular calcium regulation, NF-κB), and a p38 mitogen-activated protein kinase (p38 MAPK)-dependent mechanism (Kim et al., 2006).

Quercetin, a flavonoid aglycone, functions extensively as a major active compound in anti-allergic supplements. Both transcriptional and translational levels of AR-related mediators can be suppressed by quercetin both *in vitro* and *in vivo*. In IL-4-induced human nasal epithelial cells (HNEpCs), nitric oxide (NO) production, inducible nitric oxide synthase (iNOS) mRNA expression, and signal transducer and activator of transcription 6 (STAT6) activation are all attenuated (Ebihara et al., 2018; Edo et al., 2018). Quercetin has the ability to suppress pro-inflammatory cytokines and inflammatory inducers such as cyclooxygenase-2 (COX-2), vasoactive intestinal peptide (VIP), substance P, calcitonin gene-related peptide (CGRP), nerve growth factor (NGF), and HIR gene upregulation in animal models of antigen-induced AR (Mlcek et al., 2016). In addition, periostin, a novel marker of respiratory inflammation, and thioredoxin, an antioxidant enzyme, were found to be moderated by quercetin, resulting in an improvement in AR clinical condition (Irie et al., 2016; Edo et al., 2018). The effects of quercetin were also revealed in the clinical setting in which the combination of herbs [quercetin, *Perilla frutescens* (L.) Britton, and vitamin D3] known as “Lertal^®^” was effective in relieving the overall symptoms of AR and reducing the use of anti-allergic drugs without additional adverse effects (Thornhill and Kelly, 2000).

Other active compounds in Triphala are ascorbic acid (vitamin C) and kaempferol. In particular, the plasma level of ascorbic acid has been found to be reduced in allergic-related diseases in which patients with allergy-related respiratory and cutaneous symptoms can derive benefits after receiving an exogenous vitamin C treatment (Vollbracht et al., 2018). One study found that vitamin C in combination with exercise potentially reduced inflammatory cytokines in nasal secretion, reduced malondialdehyde (MDA), an important oxidative stress marker, and exhibited improvement in physiological function in rhinitis patients (Seo et al., 2013; Tongtako et al., 2018). Kaempferol moderates several kinds of cytokines and inflammatory markers in both the eosinophil cell line and the mouse model of OVA induction, leading to a reduction in AR-related inflammation (Oh et al., 2013).

Considering the beneficial aspect of Triphala for manipulating the pathophysiology of AR, specific convincing compounds (shown in Table 2.) are likely to be major responsible molecules since many previous studies have revealed their pharmacological activities specifically for physiological processes related to airway inflammation and AR development.

**TABLE 2:** Plant’s constituents in Triphala exhibiting potential benefits in alleviating AR symptoms.

Compound	Mechanism of action	Potential benefits in AR
Quercetin	Suppresses pro-inflammatory cytokines and inflammatory inducers such as COX-2, VIP, substance P, CGRP, NGF, and HIR gene upregulation in animal models of antigen-induced AR (Mlcek et al., 2016)	Reduction of the release of histamine, inflammatory cytokines, and chemokines Reduction of nasal symptoms
	Reduces periostin production from IL-4-induced HNEpC through inhibition of signal transducer and activator of STAT6 activation and reduces RANTES and eotaxin production in periostin-stimulated HNEpC through suppression of activation of NF-κB p65 (Irie et al., 2016)	
	Reduces NO production from nasal epithelial cells after IL-4 stimulation (Ebihara et al., 2018)	
	Reduces neuropeptide production in nasal lavage fluids induced by TDI nasal challenge in rat model (Kashiwabara et al., 2016)	
	Reduces eosinophil count in nasal mucosa in rat model of AR (Sagit et al., 2017)	
	Inhibits expression of inflammatory cytokines in stimulated human mast cell line (Min et al., 2007)	
	Inhibits the release of histamine, LTs, PGD2, and GM-CSF from human cultured mast cell (Kimata et al., 2000)	
	Inhibits the production of IL-5 and IL-13 and increases IFN-γ production from CD4^+^ T cells induced by IL-4 stimulation (modulate Th1/Th2 balance) (Tanaka et al., 2020)	
	Increases thioredoxin levels in H_2_O_2_-induced nasal epithelial cells and in nasal lavage fluid of OVA-sensitized mice and decreases nasal symptoms (Edo et al., 2018)	
	Reduces histamine- and PMA-induced up-regulation of H1R gene expression in HeLa cells (Hattori et al., 2013)	
	Reduces secretion of allergic mediators in RBL-2H3 cells, decreases CD23 mRNA expression and p38 MAPK activation in IL-4 stimulated Caco-2 cells, and suppresses IgE-OVA-induced ERK activation and chemokine release (Lee et al., 2010)	
Kaempferol	Moderates several kinds of cytokines and inflammatory markers in both the eosinophil cell line and the OVA-induced AR mouse model (Oh et al., 2013)	Reduction of the release of histamine, inflammatory cytokines, and chemokines
	Inhibits IgE-mediated anaphylaxis *in vivo* and in human mast cells, reduces serum concentrations of histamine, TNF-α, IL-8, and monocyte chemo-attractant protein-1 (Cao et al., 2020)	Reduction of allergic reaction
	Reduces serum histamine and COX-2 protein expression in lung tissues in OVA-sensitized mice (Kang et al., 2018)	
	Reduces inflammatory cells in blood sample of BSA-challenged asthmatic mice and inhibits airway wall thickening through disturbing Syk-PLCγ signaling and PKCμ-ERK-cPLA2-COX2 signaling in antigen-exposed mast cells (Shin et al., 2015)	
	Reduces LPS-induced epithelial eotaxin-1 expression, decreases TNF-α-induced eosinophil recruitment, reduces epithelial inflammation, and reduces allergic and inflammatory airway in OVA-challenged mice through disturbing NF-κB signaling (Gong et al., 2012)	
	Inhibits IL-4 activation of STAT6 through type I receptors expressing JAK3 (Cortes et al., 2007)	
	Reduces fibrotic airway remodeling via bronchial epithelial-to-mesenchymal transition by modulating PAR1 activation (Gong et al., 2014)	
	Reduces secretion histamine in IgE-stimulated RBL-2H3 cells and suppressed mast-cell-dependent PCA in IgE-sensitized mice (Kim et al., 2014)	
	Suppresses LPS-induced Th1, Th2, and neutrophil-related chemokines in THP-1 cells (Huang et al., 2010)	
	Reduces secretion of allergic mediators in RBL-2H3 cells, decreases CD23 mRNA expression and p38 MAPK activation in IL-4 stimulated Caco-2 cells, and suppresses IgE-OVA-induced ERK activation and chemokine release (Lee et al., 2010)	
Ellagic acid	Accelerates the resolution of allergic airways inflammation in mouse model of ovalbumin-induced asthma and reduces total leukocytes and eosinophils numbers in the BALF, the mucus production, and lung inflammation in part by reducing IL-5 concentration, EPO activity, and P-selectin expression (De Freitas Alves et al., 2013)	Clearance of allergic airways inflammation and asthma
	Reduces the vascular permeability changes, reduces IL-6, increases levels of IL-10, reduces neutrophil recruitment to the BALF, and reduces COX-2-induced exacerbation of inflammation (Cornélio Favarin et al., 2013)	
	Inhibits anti-DNP IgE-mediated passive cutaneous anaphylaxis, reduces histamine release and the expression and secretion of pro-inflammatory cytokines, such as TNF-α and IL-6, and reduces activation of NF-κB (Choi and Yan, 2009)	
	Antioxidant (free radical scavenging activity) (Ríos et al., 2018)	
Gallic acid	Alleviates nasal inflammation in a mouse model of OVA-induced AR (Fan et al., 2019)	Alleviation of nasal symptoms caused by inflammatory cytokines and autacoids
	Inhibits of pro-inflammatory cytokines and histamine release (Kim et al., 2006)	Reduction of oxidative stress in AR and may suppress exacerbation of AR-related symptoms
	Suppresses AR-related mediators both *in vitro* and *in vivo* (Ebihara et al., 2018; Edo et al., 2018)	
	Suppresses pro-inflammatory cytokines and inflammatory inducers (Mlcek et al., 2016)	
	Reduces markers of respiratory inflammation (Irie et al., 2016; Edo et al., 2018)	
	Inhibits IL-33-mediated ILC2 activation and subsequent Th2 cytokine release via downregulation of the MyD88/NF-κB signaling pathway in ovalbumin-induced asthma in mice (Wang et al., 2018)	
	Antioxidant (Roidoung et al., 2016)	
Vitamin C	Reduces MDA, an important oxidative stress marker (Seo et al., 2013; Tongtako et al., 2018)	Reduction of oxidative stress in AR and may suppress exacerbation of AR-related symptoms
	Combined with exercise helps increase PNIF, reduce rhinitis symptoms, nasal blood flow, and MDA levels, decreases nasal secretion IL-4, and increase nasal secretion IL-2 levels (Tongtako et al., 2018)	Reduce rhinitis symptoms and other nasal symptoms
	Less decline in pulmonary function when compared to placebo in crossover RCT (Forte et al., 2018)	
	Reduces eosinophil infiltration and increases ratio of IFN-γ/IL-5 cytokines in bronchoalveolar lavage fluid in OVA-challenged mouse model (Chang et al., 2009)	
Chebulinic acid	A strong antioxidant (Lee et al., 2007) Inhibits influenza viral neuraminidase (Li et al., 2020)	Reduction of oxidative stress in AR and may suppress exacerbation of AR-related symptoms
	DNA gyrase inhibitors (Khan et al., 2018)	Alleviation of symptoms resulted from viral and bacterial infection in AR patients
Chebulagic acid	Inhibits enterovirus 71 replication, inhibits influenza viral neuraminidase (Yang et al., 2013)	Alleviation of symptoms resulted from viral infection in AR patients
	Reduces onset and progression of collagen-induced arthritis in mice (reduces serum levels of total and anti-collagen IgG, IL-10, and IL-6 but increases TGF-β) (Lee et al., 2005)	Reduction of inflammatory signaling in the airway
	Inhibits the LPS-induced expression of TNF-α and IL-1β in endothelial cells (Liu et al., 2015)	
	Inhibits COX-2 and 5-LOX enzymes (Reddy et al., 2009; Athira et al., 2013)	
Emblicanin	Possesses strong free radical-scavenging activity (>gallic acid > ellagic acid > ascorbic acid) (Pozharitskaya et al., 2007)	Reduction of oxidative stress in AR and may suppress exacerbation of AR-related symptoms

BALF, bronchoalveolar lavage fluid; cPLA2, cytosolic phospholipase A2; DNA, deoxyribonucleic acid; DNP, dinitrophenyl; EPO, eosinophil peroxidase; ERK, extra signal-regulated protein kinase; GM-CSF, granulocyte-macrophage colony-stimulating factor; H1R, histamine H1 receptor; THP-1, human monocyte cell line; H_2_O_2_, hydrogen peroxide; LTs, leukotrienes; LPS, lipopolysaccharide; LOX, lipoxygenases; PAC, passive cutaneous anaphylaxis; PNIF, peak nasal inspiratory flow; PMA, phorbol-12-myristate-13-acetate; PLCγ, phospholipase Cγ; PAR-1, protease-activated receptor-1; PKCμ, protein kinase Cμ; RCT, randomized clinical trial; RANTES, regulated on activation; normal T-cell expressed and secreted; TGF-β, transforming growth factor-β; TBI, toluene 2,4-diisocyanate.

### Triphala: Antioxidative Effects

All living cells of organisms are a major source of ROS. Moreover, other exogenous sources of oxidative stress can also induce an exacerbation of AR symptoms. For example, 1) urban pollutants aggravate nasal inflammation and AR-related symptoms, 2) pollen grains that contain endogenous reduced nicotinamide adenine dinucleotide phosphate (NADPH) oxidase damage airway epithelial cells and trigger granulocyte recruitment (Boldogh et al., 2005). However, our bodies have sophisticated antioxidant defense mechanisms, for example, enzymatic mechanisms, including glutathione-S-transferase (GST), superoxide dismutase (SOD), catalase (CAT), glutathione peroxidase (GPx), as well as non-enzymatic mechanisms involving glutathione, ascorbate, urate, *a*-tocopherol, bilirubin, lipoic acid, transferrin, and albumin, in order to prevent overwhelming by and accumulation of oxidative stress (Frei et al., 1988; Adwas et al., 2019). The imbalance between ROS production and endogenous antioxidant defense leads to an exhaustion of oxidative stress markers such as NO, MDA, and nitrite/nitrate (Birben et al., 2012; Ayala et al., 2014; Nadif et al., 2014).

Oxidative stress is now considered to be one of the markers of the pathogenesis of AR, asthma, and chronic obstructive pulmonary disease (COPD). Epidemiological and clinical studies of AR have found that eroallergen and pollutant co-exposure can induce immunological effects, leading to recruitment of inflammatory cells, cytokines, chemokines, and finally AR-related symptoms (Naclerio et al., 2020). Ozone exposure was also found to exacerbate antigen-induced rhinitis, nasal symptoms such as rhinorrhea, sneezing, nasal hyperresponsiveness, and eosinophil infiltration in guinea pigs (Iijima et al., 2001). An increase in plasma total oxidant status (TOS) and a decrease in the plasma antioxidative enzyme paraoxonase (PON-1) have both been detected in children with persistent AR, and these two factors were correlated with their nasal symptoms. TOS and PON-1 may serve as disease markers in children with AR (Ozkaya et al., 2013). Therefore, any treatments that can attenuate this increase in oxidants and prevent the decrease in endogenous antioxidants may help improve AR-related symptoms and can be considered a new strategy for treating AR.

In recent decades, several studies have examined and described the antioxidative properties of Triphala, as shown in Table 3. Even with different extraction methods, Triphala retains its ability to scavenge free radicals in both *in vitro* and *in vivo* models. For this aspect, several studies reported the activity of Triphala by using chemical assays in which the results do not directly represent the antioxidative properties of Triphala in the actual physiological condition and cannot guarantee its intracellular antioxidant activities (Naik et al., 2006; Liu et al., 2008; Mehrotra et al., 2011; Babu et al., 2013; Gajendra et al., 2016; Varma et al., 2016) However, screening by using such *in vitro* chemical assays provide beneficial information related to potential antioxidant activities of Triphala, which may be further verified in the cell models or animal models. An enrichment of polyphenolic compounds such as phenolic acids, flavonoids, and tannins is considered to be one of the most important oxidant defensive mechanisms of Triphala (Rice-Evans et al., 1997; Francenia Santos-Sánchez et al., 2019). Thus, quantification of total phenolic content using gallic acid as a standard is necessary (Chandra et al., 2014). Parveen et al. discovered that the methanolic extract of Triphala and its constituents showed a more intense chromogenic reaction than aqueous extracts, and this result supports its greater free radical scavenging ability (Parveen et al., 2018). Interestingly, Triphala possesses radical scavenging effects by reducing the concentrations of oxidative stress molecules and products, and it also works to increase the number of antioxidant enzymes present (Figure 2) (Gupta et al., 2010). Giving Triphala in dimethylhydrazine (DMH)-induced endoplasmic reticulum (ER)-stressed mice for 35 days can prevent an increase in hepatic oxidative products, including MDA and lactate dehydrogenase (LDH), and it also helps to maintain the levels of antioxidative enzymes (GST and GSH) (Sharma and Sharma, 2011). These effects have also been found in a noise-stress induction model, with additional benefits for the restoration of plasma vitamin C and corticosterone levels (Srikumar et al., 2006). Because Triphala contains three different herbs, some researchers have tried to compare the overall ability of Triphala in terms of the potency of different herb extracts. Hazra et al. found that each herb contained individual polyphenolic profiles. The methanolic extract of *P. emblica* L. expresses higher phenolic and ascorbic contents than those of *T. chebula* Retz. and *T. bellirica* (Gaertn.) Roxb., and its scavenging ability for 2,2-diphenyl-1-picrylhydrazyl (DPPH) and peroxynitrite radicals seems to be superior. On the other hand, although *T. bellirica* (Gaertn.) Roxb. contains fewer polyphenolic compounds than the other two herbs, it still has a greater effect in reducing the number of superoxide, nitric oxide, and hydroxyl radicals (Hazra et al., 2010). However, different sample sources, herbal proportions, and extraction methods can cause variations in the medicinal profiles (Liu et al., 2008; Parveen et al., 2018). Therefore, it is important to carry out herb qualification and characterization before examining the effects of these herbs or permitting their consumption by humans.

**TABLE 3:** Triphala and its constituents with anti-oxidative properties.

Herbs	Extracts	Models		Results	References
		*In vitro*	*In vivo*		
Triphala	50% ethanol	Bleomycin and paraquat- induced ROS in HeLa cells		↓ROS levels in bleomycin induction (Triphala > T3 > T2 > T1), in paraquat induction (T1 > Triphala > T2 > T3) (dose = diluted at 3 × 10^–5^)	(Takauji et al., 2016)
*P. emblica* L. (T1)					
*T. chebula* retz. (T2)					
*T. bellirica* (gaertn.) roxb. (T3)					
Triphala	n/a (powder mixed with diet)		Mice DMH-induced ER stress in mouse liver. (5% w/w mixed with diet for 14 days; negative control: normal diet)	↓Hepatic oxidative stress (↓MDA and LDH) ↑Anti-oxidative enzymes (↑GSH and GST)	(Sharma and Sharma, 2011)
Triphala	Mixed with normal saline		Rat LPO, SOD, CAT, and GPx levels in noise-induced stress rats (extract = 1 g/kg, p.o. for 48 days; negative control: Saline)	↓MDA and corticosterone while ↑SOD, GPx, and vitamin C level in plasma and spleen (MAC = 1 g/kg)	(Srikumar et al., 2006)
				↑CAT in spleen (MAC = 1 g/kg)	
*P. emblica* L. (T1)	70% methanol		Mice SOD, CAT, GST, GSH in liver (extract = 10, 50, and100 mg/kg, p.o. for 7 days) negative control: normal saline	↑SOD, CAT, GST, and GSH content in liver (MAC = 10 mg/kg)	(Hazra et al., 2010)
*T. chebula* retz. (T2)					
*T. bellirica* (gaertn.) Roxb.(T3)					
*P. emblica* L	Water	H_2_O_2_- induced U937 human myeloleukemic model		↓ROS production in H_2_O_2_-induced human myeloleukemic U937 cells (IC50 = 295.0 μg/ml)	(Charoenteeraboon et al., 2010)
		Positive control: Gallic acid, trolox			
*T. chebula* retz	Distilled water		Young and aged rats (extract = 200 mg/kg, p.o. for 4 weeks; negative control: sterile water)	↑Enzymatic (MnSOD, CAT, GR, GST, GSH, G6PDH) and non-enzymatic antioxidants (GPx, vitamin E, and vitamin C levels) in liver and kidney of aged rats	(Mahesh et al., 2009)
				↓Oxidative stress markers (MDA, PCO, LF, XO) in liver and kidney of aged rats	
				↑Enzymatic (GST, G6PD) and non-enzymatic antioxidants (GST) as well as ↓Oxidative stress marker (MDA, PCO, XO) in liver of young rats	

DMH, 1,2-dimethylhydrazinedihydrochloride; ABTS^·−^, 2,2′-azino-bis(3-ethylbenzthiazoline-6-sulphonic acid); IC50, 50% inhibitory concentration; ER, endoplasmic reticulum; EDTA, ethylenediaminetetraacetic acid; FRAP, ferric reducing ability of plasma; G6PD, glucose-6-phosphate dehydrogenase; GR, glutathione reductase; HDF, human dermal fibroblast; H_2_O_2_-, hydrogen peroxide; ^·^OH, hydroxyl radical; HOCl, hypochlorous acid; LPO, lipid peroxidation; LF, lipofusin; Mn-SOD, manganese superoxide dismutase; MAC, minimal active concentration; ^·^NO, nitric oxide; ONOO^·−^, peroxynitrite; PCO, protein carbonyl; RBC, red blood cell; Roxb.; GSH, reduced glutathione; TEAC, Trolox equivalent antioxidant concentration; ^1^O_2_, singlet oxygen; O_2_^·−^, superoxide anion radical; XO, xanthine oxidase.

### Triphala: Anti-Inflammation

Inflammation plays an important role during AR progression (Gelfand, 2004). Several kinds of inflammatory cytokines have been found to be increased in AR patients in both the early and late phases of allergen-induced inflammation. IgE-mediated mast cell degranulation in the early phase is an initial factor in inducing Th2 and T helper cell 17 (Th17) differentiation (Iwakura et al., 2008; Mukai et al., 2018). Cytokines, such as IL-4, IL-13, and IL-17, released by these T cells possess an ability to aggravate nasal mucosa inflammation and disturb the intact mucosal barrier (Wise et al., 2014; Ramezanpour et al., 2016). Many studies in both *in vitro* and *in vivo* models have revealed the marked benefits of inflammatory modulation of AR conditions by using various anti-inflammatory approaches (Holgate et al., 2005; Canonica and Baiardini, 2006). An *in vitro* IL-4 and IL-13 antagonizing treatment and an *in vivo* anti-IL-4 treatment in mice were found to prevent epithelial barrier disruption (Steelant et al., 2018). Suppression of IL-4, TNF-α, IgE, and eosinophil levels in serum significantly alleviated symptoms and improved QoL in AR patients (Lv et al., 2018). Additionally, the strategy of upregulating anti-inflammatory signaling pathways, nuclear factor erythoid 2-related factor 2/heme oxygenase-1 (Nrf2/HO-1), and suppression of toll-like receptor-myeloid differentiation primary response 88-nuclear factor kappa B (TLR-MyD88-NF- κB) are all promising strategies for relieving nasal inflammation and improving airway epithelial barrier integrity (Bui et al., 2020).

Regardless of the prominent effects of a single herb, the polyherbal Triphala formulation possesses anti-inflammatory activity by acting through several mechanisms (Figure 2; Table 4). Using different extraction methodologies, the herbs retain their ability to reduce both acute and chronic inflammation. For example, at a dose of 100–200 mg/kg, Triphala was shown to be effective in reducing inflammation in carrageenan- and ethyl phenylpropiolate (EPP)-induced paw edema, which are both used as acute inflammatory models (Prabu et al., 2008). Similarly, chronic inflammation in a granuloma formation induced by a cotton pellet was also reduced by a Triphala pretreatment (Prabu et al., 2008). Sireeratawong et al. reported that even though the animal received a high dose of Triphala water extracted at 1,200 mg/kg, the granuloma formation was not reduced by the cotton pellet induction model (Sireeratawong et al., 2013). On the other hand, another study using a similar induction model found that the methanolic extract was beneficial, especially at lower doses (100–200 mg/kg) (Prabu et al., 2008). These results suggest that the methanolic extract might possess higher potency than the water extract, hence further comparisons of the active compounds identified in each extract are needed in order to address this point.

**TABLE 4:** Triphala and its constituents with anti-inflammatory properties.

Herbs	Extracts	Models		Results	References
		*In vitro*	*In vivo*		
Triphala	Water		Mice	↓paw edema	(Rasool and Sabina, 2007)
			CFA-induced arthritis (extract = 1 g/kg, p.o. for 8 days)	↓lysosomal enzymes (acid phosphatase, E-glucuronidase, N-acetyl glucosaminidase, and E-galactosidase) in plasma, liver, and spleen	
			Positive control: indomethacin (3 mg/kg, p.o. for 8 days)	↓protein-bound carbohydrates (hexose, hexosamine, hexuronic acid, fucose, and sialic acid) in liver and spleen	
				↓marker enzymes (AST, ALT, and ALP) in plasma, liver, and spleen	
Triphala	Methanol		Rats	↓paw edema (MAC = 100 mg/kg)	(Prabu et al., 2008)
			Carrageenan-induced edema (extract = 100 and 200 mg/kg, p.o. single dose)	↓wet and dried weight of cotton pellet (MAC = 200 mg/kg)	
			Cotton pellet induced granuloma (extract = 100 and 200 mg/kg, p.o. for 7 days; negative control: 1% tween 80; positive control: indomethacin = 10 mg/kg, p.o. for 7 days)		
Triphala	Water		Rats	↓EPP-induced ear edema	(Sireeratawong et al., 2013)
			EPP and AA-induced ear edema (extract = 4 mg/ear, topical application; negative control: Mixture of dimethysulfoxide and acetone, 1:1; positive control: phenylbutazone = 1 mg/ear, phenidone = 2 mg/ear)	↔ AA-induced ear edema	
			Carrageenan-induced paw edema (extract = 300–200 mg/kg, p.o.; negative control: distilled water; positive control: aspirin = 300 mg/kg)	↓carrageenan-induced paw edema (MAC = 300 mg/kg)	
			Cotton pellet-induced granuloma formation (extract = 1,200 mg/kg, p.o. for 7 days; negative control: distilled water; positive control: aspirin = 300 mg/kg, prednisolone = 5 mg/kg)	↔ granuloma formation	
Triphala	Water		Rats	↓lipid peroxidation, glycoproteins (hexose, hexosamine, and sialic acid), and lysosomal enzymes (acid phosphatase, β-galactosidase, N-acetyl β-glucosaminidase, and cathepsin-D) in paw tissues	(Kalaiselvan and Rasool, 2015)
			CFA-induced arthritis (extract = 100 mg/kg, i.p. for 7 days; negative control: saline; positive control: indomethacin = 3 mg/kg, i.p.)	↑SOD, CAT, GPx, GST, and GSH in paw tissues	
				↓TNF-α, IL-1β, VEGF, MCP-1, and PGE2 levels in serum and paw tissues	
Triphala	Distilled water	LPS stimulated RAW 246.7 macrophage (extract = 100–300 μg/ml)	Rats	↓TNF-α, IL-1β, IL-6, MCP-1, VEGF, NO, PGE2 levels (MAC = 200 μg/ml)	(Kalaiselvan and Rasool, 2016)
			CFA-induced arthritis extract = 100 mg/kg, i.p. for 7 days; negative control: Saline; positive control: indomethacin = 3 mg/kg, i.p. for 7 days)	↓lysosomal enzymes [β-galactosidase, N-acetyl β-glucosaminidase, cathepsin-D (MAC = 100 μg/ml), and acid phosphatase (MAC = 300 μg/ml)]	
				↓*TNF-α, IL-1β, IL-6, MCP-1, NF-kBp65, COX-2* and ↑*HO* mRNA expression (MAC = 200 μg/ml)	
				↓NF-kBp65 and pNF-kBp65 protein expression (MAC = 200 μg/ml)	
				↓overexpressed TNF-α, IL-17, iNOS, COX-2 (MAC = 200 μg/ml)	
				↓free radical formation (MAC = 100 μg/ml)	
				↓p-NF kB p65, IL-17, COX-2 and RANKL protein expression in paw tissues	
Triphala (AIE)	Ethanol	TNF-α induced inflammation in retinal choroid microvascular endothelial cells (RF/6 A) (AIE = 1–50 μg/ml, CA, CI and GA = 1–100 μg/ml)	*Ex vivo*: TNF-α induced angiogenesis in chick chorioallantoic membrane (AlE = 25 μg, CA = 25 μM, CI = 25 μM, GA = 100 μM)	↓MMP-9 expression (MAC of AIE, CA and CI = 10 μg/ml, GA = 50 μg/ml)	(Shanmuganathan and Angayarkanni, 2018)
Chebulagic acid (CA)		*In silico* docking study: binding to TNF-α-receptor-1		↓tube formation, chemotaxis, endothelial cell proliferation (MAC of AIE, CA and CI = 10 μg/ml, GA = 100 μg/ml)	
Chebulinic acid (CI)				↓IL-6, IL-8, and MCP-1 expression (MAC of AIE = 100 μg/ml, CA and CI = 10 μg/ml, GA = 50 μg/ml)	
Gallic acid (GA)				↓phosphorylation of p38, ERK and NF-kB (MAC of AIE, CA and CI = 10 μg/ml, GA = 100 μg/ml)	
				Binding energy of CA = 6.4 kcal/mol, CI = 7.7 kcal/mol, GA = 6.3 kcal/mol	
				↓angiogenesis (MAC of AIE, CA and CI = 25 μg/ml, GA = 100 μg/ml)	
*P. emblica* L	80% methanol		Rats	↓serum LDH (MAC = 100 mg/kg)	(Deshmukh et al., 2010)
			Acetic acid induced colitis (extract = 100 and 200 mg/kg, p.o. for 7 days; positive control: sulfasalazine = 500 mg/kg, p.o for 3 days)	↓macroscopic inflammation scores and wet weight of colonic segments (MAC = 200 mg/kg)	
*P. emblica* L	Hydroalcohol		Rats	↓lipid peroxidation in brain (MAC = 300 mg/kg)	(Golechha et al., 2011)
			KA-induced seizures (extract = 300, 500, and 700 mg/kg, i.p. for 7 days; negative control: Normal saline, i.p.)	↑brain GSH levels (MAC = 500 mg/kg)	
				↓TNF-α in brain (MAC = 300 mg/kg)	
*P. emblica* L	70% ethanol		Rats	Both FPEO and BREO	(Muthuraman et al., 2011)
	free (FPEO) and bounded (BREO) phenolic compounds		Carrageenan-induced paw edema (FPEO and BREO = 20 and 40 mg/kg, p.o.; positive control: diclofenac sodium = 12.5 mg/kg, p.o.; negative control: 1% of carboxy methyl cellulose = 1 ml, p.o.)	↓paw edema (MAC = 40 mg/kg)	
			Cotton pellet-induced granuloma formation (extract = 20 and 40 mg/kg, p.o. for 16 days; negative control: 1% of carboxy methyl cellulose = 1 ml, p.o.; positive control: diclofenac = 12.5 mg/kg, p.o.)	↓granulomatous tissue mass (MAC = 40 mg/kg)	
				↓lipid peroxidation, myeloperoxidase activity, plasma extravasaion levels and ↑GSH level (MAC = 40 mg/kg)	
*P. emblica* L	Commercial fruit extract (patented enzymatic process)	LPS-induced inflammation in HUVEC (extract = 0–100 μg/ml)	Rats	↓adhesion of THP-1 cells (MAC = 1 μg/ml)	(Pradyumna Rao et al., 2013)
			LPS-induced endotoxemia (50 mg/kg, p.o.; negative control: PBS = 50 mg/kg, p.o.)	↓E-selectin mRNA expression (MAC = 30 μg/ml)	
				↓serum TNF-α and IL-6 levels (MAC = 50 mg/kg)	
*P. emblica* L	Hydro-alcohol		Rats	↓paw edema (MAC = 300 mg/kg)	(Golechha et al., 2014)
			Carrageenan (positive control: indomethacin 10 mg/kg, p.o.), histamine (positive control: CPM 3 mg/kg, p.o.), serotonin (positive control: CPH 3 mg/kg, p.o.), and PGE2 (positive control: PBZ 100 mg/kg, p.o.)-induced hind paw edema (extract = 300, 500, and 700 mg/kg, i.p.; negative control: saline = 1 ml/kg, i.p.)	↑GSH, catalase, and SOD level in paw tissues (MAC = 500 mg/kg)	
			Cotton pellet-induced granuloma formation (extract = 300, 500, and 700 mg/kg, i.p. for 7 days; negative control: Saline = 1 ml/kg, i.p.; positive control: indomethacin = 10 mg/kg, p.o.)	↓MDA level in paw tissues (MAC = 300 mg/kg)	
				↓granulomatous tissue mass (MAC = 300 mg/kg)	
*P. emblica* L	Methanol		Rats	↓paw edema (MAC = 200 mg/kg)	(Middha et al., 2015)
			Carrageenan-induced paw edema (extract = 200 and 400 mg/kg; positive control: diclofenac = 10 mg/kg)	↓IL-1β, TNF-α levels (MAC = 200 mg/kg)	
*P. emblica* L	Ethyl acetate fraction		Mice	↓serum TNF-α, IL-1β, IL-6 (MAC = 500 mg/kg)	(Singh et al., 2015)
			Arsenic-induced inflammation (extract = 500 mg/kg, p.o. for 30 days; negative control: 2% gum acacia)	Restore T cells and B cells	
				Reverse thymus and spleen weight	
*T. chebula* retz	50% methanol		Rats	↓joint swelling (MAC = 40 mg/kg)	(Nair et al., 2010)
			Formaldehyde-induced arthritis (extract = 20, 40, and 80 mg/kg, p.o. for 10 days)	↓serum TNF-α (MAC = 40 mg/kg)	
			CFA-induced arthritis (extract = 20, 40, and 80 mg/kg, p.o. for 21 days; positive control: indomethacin = 3 mg/kg)	↓synovial expression of IL-1β, IL-6, and TNFR1 (maximum at 80 mg/kg)	
				↓joint swelling (MAC = 20 mg/kg)	
*T. chebula* retz	50% methanol		Rats	↓paw edema (MAC = 80 mg/kg)	(Nair et al., 2012)
			Carrageenan-induced paw edema (extract = 20, 40, and 80 mg/kg, p.o.)	↓dry granuloma weight (MAC = 40 mg/kg)	
			Cotton pellet-induced granuloma formation (extract = 20, 40, and 80 mg/kg, p.o. for 6 days)	↓TNF-α, IL-6 and IL-β levels (MAC = 80 mg/kg)	
			CFA-stimulated peritoneal macrophages (extract = 80 mg/kg, p.o. for 6 days; negative control; 1% gum acacia; positive control: indomethacin = 3 mg/kg)	↓TNF-Rl expression on peritoneal macropharge (MAC = 80 mg/kg)	
*T. chebula* retz	95% ethanol		Mice	↓ear thickness (MAC = 100 mg/kg)	(Sukakul et al., 2013)
			Croton oil-induced ear dermatitis (extract = 100 mg/ml, topical application)	↓ear plug weight (MAC = 100 mg/kg)	
*T. chebula* retz	70% ethanol	Human RBC stablization method (extract = 50, 100, 250, 500 μg/ml)	Rats	↓MDA formation (MAC = 50 mg/kg)	(Bag et al., 2013)
			Carrageenan-induced high paw edema (extract = 50, 100, 250, and 500 mg/kg p.o.; negative control: normal saline; positive control: indomethacin 5 mg/kg, p.o.)	↓paw edema (MAC = 50 mg/kg)	
				↑human RBC membrane stability (MAC = 50 μg/ml)	
*T. chebula* retz	80% ethanol		Rats	↓paw edema (MAC = 300 mg/kg)	(Ibne Jami et al., 2014)
			Carrageenan-induced paw edema (extract = 300 mg/kg; negative control: normal saline; positive control: diclofenac 100 mg/kg)		
*T. chebula* retz	100% methanol	LPS-stimulated mouse macrophage cells (RAW 264.7) (compounds = 10, 50 μM; positive control: parthenolide 10 μM, indomethacin 10 μM)		↓NO production	(Yang et al., 2014)
				chebulinic acid (MAC = 50 μM)	
				2,3,6-tri-O-galloyl-B-d-glucose (MAC = 50 μM)	
				arjunuc acid (MAC = 50 μM)	
				arjulonic acid (MAC = 50 μM)	
				↓expression of iNOS (MAC = 10 μM) and COX-2 (MAC = 50 μM)	
*T. chebula* retz	Water		Rats	↓EPP-induced ear edema (MAC = 1 mg/ear)	(Sireeratawong et al., 2014)
			EPP- and AA-induced ear edema (extract = 1, 2, 4 mg/20 μL/ear; positive control: phenylbutazone, phenidone 1 and 2 mg/ear)	↔ AA-induced ear edema	
			Carrageenan-induced paw edema (extract = 150, 300, and 600 mg/kg; negative control: normal saline; positive control: Aspirin 300 mg/kg)	↓paw edema (MAC = 1 mg/kg)	
			Cotton pellet-induced granuloma formation (extract = 600 mg/kg, p.o. for 7 days; positive control: Aspirin 300 mg/kg, prednisolone 5 mg/kg)	↔ granulomatous tissue mass	
*T. chebula* retz	Methanol		Rats	↓paw edema (MAC = 100 mg/kg)	(Kirubanandan et al., 2015)
			Carrageenan-induced paw edema (extract = 100 and 200 mg/kg, p.o.)	↓granulomatous tissue (MAC = 200 mg/kg)	
			Cotton pellet-induced granuloma formation in rats (extract = 100 and 200 mg/kg, p.o. for 7 days; negative control: 1% Tween80 2 ml/kg, p.o; positive control: indomethacin” 10 mg/kg, p.o.)		
*T. chebula* retz	*Terminalia* chebulanin (polyphenolic compound from fruit)	M5 (a cocktail of IL-1α, IL-17A, IL-22, oncostatin M, and TNFα)-induced HaCaT cells proliferation (extractt = 10 μg/ml)	Mice	↓TBARS content, ROS production	(An et al., 2016)
			IMQ-induced psoriatic skin lesion (extract = 50 mg/kg, intragastric; negative control: vehicle vaseline cream)	↑GSH level (MAC = 10 μg/ml)	
				↓*TNFα, IL-17A, IL-23b, MMP-9, cyclinD, cyclinE* mRNA expression (MAC = 10 μg/ml)	
				↓overexpressed p65 NF-κB expression in tissues and cells (MAC = 10 μg/ml)	
				↓skin leison through heme oxygenase-I (MAC = 50 mg/kg)	
				↓TNFα, IL-17 A and IL-23 b levels in serum and skin (MAC = 50 mg/kg)	
				↑HO-1 expression (MAC = 50 mg/kg)	
*T. chebula* retz	Water		Osteoarthritic dogs (extract = 500 mg p.o. bid for 150 days; negative control: placebo capsule)	↓pain upon limb manipulation significance at 90 days ↓ESR significance at 120 days	(Murdock, 2015)
*T. chebula* retz	70% ethanol	LPS-induced primary microglia cells isolated from mice (extract = 5,10,20,40.80 μg/ml)		↓TNF-α, IL-1β, IL-6, COX-2, PGE-2, NO concentration (MAC = 20 μg/ml)	(Rahimi et al., 2018)
				↓*TNF-α, IL-1β, IL-6, COX-2, iNOS* mRNA expression (MAC = 20 μg/ml)	
				↑*Arg-1* mRNA expression (dose dependence) (MAC = 20 μg/ml)	
				↑urea production (MAC = 40 μg/ml)	
*T. bellirica* (gaertn.) roxb	70% aqueous acetone	Radical scavenging activity		↓ROS generation in LPS stimulated RAW 264.7 macrophage cells (MAC = 100 μg/ml)	(Jayesh et al., 2018)
		LPS-induced macrophage RAW 264.7 cells (extract = 25, 50, 100 μg/ml; positive control: diclofenac sodium)		↓total COX activity, MPO, total 5-LOX activity (MAC = 25 μg/ml)	
				↓*TNF-α*, *IL-6* and *COX-2* mRNA expression (MAC = 25 μg/ml)	
				↓ROS production (MAC = 100 μg/ml)	
*T. bellirica* (gaertn.) roxb	Water	LPS- or palmitic acid-induced macrophage RAW 264.7 cell (extract = 100, 200, 400 μg/ml; negative control: Distilled water; positive control: GA = 46 μg/kg, EA = 1.6 μg/kg)	LPS-shock model mice (extract = 400 mg/kg, p.o.; negative control: Distilled water)	↓*TNF-α*, *IL-1β*, *IL-6*, *MCP-1*, *CCL-2*, *MSR, NOS2* mRNA expression (MAC = 400 μg/ml)	(Tanaka et al., 2018)
				↓SR-A protein expression	
				↓ROS production (MAC = 100 μg/ml)	
				↓NF-κB nuclear translocation, ↓phosphorylation of NF-κB p65, p38, JNK, and ERK (MAC = 400 μg/ml)	
				Activate Akt/AMPK/Nrf2 pathway (MAC = 100 μg/ml)	
				↑mRNA expression of antioxidant enzymes (catalase, NQO1, and GCLM)	
				↓TNF-α and IL-6 mRNA expression	
				Prevent oxidative stress and inflammation in endotoxemic mice	
*T. bellirica* (gaertn.) roxb	Ethanol		Rats	↓paw edema (MAC = 100 mg/kg)	(Chauhan et al., 2018)
			Carrageenan-induced paw edema (extract = 100, 200, and 400 mg/kg; negative control: distilled water, positive control: Indomethacin 3 mg/kg)		

ALP, alanine aminotransferase; ALT, alkaline phosphatase; AA, arachidonic acid; AST, aspartate aminotransferase; *CCL-2*, *C-C motif chemokine ligand 2* gene; CPM, chlorpheniramine; SR-A, class A scavenger receptor A; CFA, complete Freund’s adjuvant; CPH, cyproheptadine; ESR, erythrocyte sedimentation rate; GCLM, glutamate-cysteine ligase modifier subunit; HUVEC, human umbilical vein endothelial cells; IMQ, imiquimod; KA, kainic acid; *MSR*, *macrophage scavenger receptor* gene; MPO, myeloperoxidase; NQO1, NADPH quinone oxidoreductase 1; PBZ, phenylbutazone; RANKL, receptor activator of nuclear kappa-B ligand; TBARS, thiobarbituric acid reactive substances; vWF, von Willebrand factor.

Most of the anti-inflammatory properties of Triphala have been reported in arthritis models. Pretreatment with the herbs significantly alleviated the inflammatory markers and enzymes associated with arthritis (Rasool and Sabina, 2007). Moreover, over-activation and overexpression of the NF-κB pathway and the production of cytokines, chemokines, and growth factors, such as TNF-α, IL-17, TNF-α, IL-1β, IL-6, monocyte chemoattractant protein-1 (MCP-1), vascular endothelial growth factor (VEGF), prostaglandin E2 (PGE2), COX-2, NO, and iNOS, were attenuated when patients were pretreated with Triphala compared with the untreated groups (Kalaiselvan and Rasool, 2016). Compound identification was partially accomplished by Shanmuganathan et al. by focusing on the TNF-α modulatory effect in which chebulinic acid, gallic acid, and the whole Triphala extract showed inhibitory characteristics on inflammation markers. These effects were mediated through the MAPK and NF-κB signaling pathways. Moreover, the authors confirmed the binding capability of these active compounds to TNF-α-receptor-1 by using an in silico (i.e., computer simulated) approach (Shanmuganathan and Angayarkanni, 2018).

Although the effect of Triphala on AR has not yet been investigated, this polyherbal recipe counteracts various kinds of inflammation, suggesting the possibility of using these herbs to treat many inflammation-related diseases, including AR. High penetrating capacity and high toxic threshold are considered to be the advantages of Triphala for developing a preferential therapeutic formulation in the future. Its highly penetrative ability is evident in different parts of various body systems, such as the effects of *P. emblica* L. in attenuating brain inflammation in epileptic models and the reduction in psoriasis-related skin inflammation by *T. chebula* Retz (Golechha et al., 2011; An et al., 2016).

Activation and translocation of NF-κB are fundamental steps for increasing inflammation-related gene expression and maintaining airway inflammation (Hart et al., 1998; Schuliga, 2015). Although no study has reported a direct effect of Triphala on airway inflammation, there is some evidence that suggests that this polyherbal recipe might be a good candidate for use in AR treatment. Triphala and its active constituents (*T. bellirica* (Gaertn.) Roxb. and *T. chebula* Retz.) exhibited an ability to decrease NF-κB activation and nuclear translocation by suppressing the phosphorylation of p38, c-Jun N-terminal kinase (JNK), and ERK and activating the Akt/AMP-activated protein kinase/nuclear factor erythoid 2-related factor (Akt/AMPK/Nrf) signaling pathway (Tanaka et al., 2018). Therefore, further studies need to be undertaken in order to explore the effects of Triphala in AR models.

### Triphala: Immunomodulation

A balance between immunostimulation and immunosuppression is important for helping our bodies maintain physiological function. In both autoimmune and inflammatory diseases, immunological imbalance occurs mainly in helper and regulatory T cells (Dejaco et al., 2006; Noack and Miossec, 2014). Especially in AR, which is known as a type I hypersensitivity condition, several immune cells are prone to activation and to be aberrant in both the sensitization and response phases (Kiboneka and Kibuule, 2019). A shift predominantly to Th2 cell responses or a lowering of the Th1/Th2 ratio has been found in several allergic diseases (Romagnani, 2004). Normally, Th1 responses result in IL-2, IFN-gamma, IgG2a, IgG2b, and IgG3 production, whereas Th2 responses lead to IL-3, IL-4, IL-5, IL-13, and IgG1 production (Gelfand, 2004; Galli et al., 2008). Most of the Th2 cytokines act as enhancers of the inflammatory process. For example, IL-3 and IL-5 affect mast cells and eosinophil proliferation and differentiation (Denburg et al., 1994; Kouro and Takatsu, 2009). IL-13 participates in the recruitment of inflammatory cells to inflammation sites by enhancing VCAM-1 expression (De Vries, 1998). An increment in IL-4, which is considered to be a major mediator in AR, can activate IgE production in B cells (Poulsen and Hummelshoj, 2007).

In herbal medicine, quercetin is a well-known candidate compound for AR-related cytokine modulation. This compound is one of the essential elements in the Triphala formulation. Quercetin has been reported to inhibit cytokines such as IL-5, RANTES, and eotaxin released by eosinophils and mast cells (Sakai-Kashiwabara and Asano, 2013). Moreover, Th1 and Th2 markers including IFN-gamma and IL-4, which are aberrantly expressed in an asthmatic model, were found to be modulated by quercetin (Park et al., 2009).

Researchers have found that Triphala and its active constituents have dual activities in relation to both immunostimulant and immunosuppressive effects that might be beneficial in AR treatment (Figure 2; Table 5). Pretreatment with Triphala (1 g/kg/day) improved neutrophil function and reduced *Pan* T and CD4^+^/CD8^+^ along with an increase in corticosteroid levels in a noise-induced stress model (Srikumar et al., 2007). Interestingly, these herbs also reduced IL-4 (Th2 cytokine) and increased IL-2 and IFN-gamma (Th1 cytokines), suggesting an influence on Th1 shifting (Srikumar et al., 2005, 2007). In nonspecific mitogen-induced T lymphocyte proliferation, the application of Triphala reduced T cell proliferation, together with a reduced complement, antibody titer, and a delayed-type hypersensitivity (DTH) response following CFA and sheep red blood cell (SRBC) induction (Sabina et al., 2009). Similar results have also been observed in a single constituent, especially with *T. chebula* Retz. Atopic lesions induced by 2,4-dinitrofluorobenzene (DNFB) were relieved following topical application of *T. chebula* Retz. extract. As well as lesion improvement, the extract attenuated atopic markers (MMP-9 and IL-31) and eosinophil infiltration in affected and adjacent areas, respectively (Nam et al., 2011). Another interesting finding was that this extract was able to shift forward the Th1 response by attenuating IL-4 and increasing IFN-gamma in an OVA-induced allergic model (Rubab and Ali, 2016). The results strongly suggest the benefits of using *T. chebula* Retz. extract for treating AR because the shifting of immunity from Th2 to Th1 is also used to define a successful response following treatment with allergen-specific immunotherapy (AIT) (Lam et al., 2020), and alteration of Th2 cytokines by reducing the Th2 response may offer a kind of protective effect in treating allergic diseases (Bosnjak et al., 2011). Moreover, upregulation of IFN-gamma in T cells is correlated with a reduction in nasal symptoms following exposure to allergens (Durham et al., 1999).

**TABLE 5:** Triphala and its constituents with immunomodulating properties.

Herbs	Models	Extracts		Results	References
		*in vitro*	*in vivo/*clinical		
Triphala	Fruit powder mixed with saline		Rats	↑Neutrophil function (adherence, phagocytic index, avidity index, NBT reduction)	(Srikumar et al., 2005)
			Noise-induced stress (extract = 1 g/kg/day for 48 days, p.o.; negative control: Saline)	↓Corticosterone level	
Triphala	Fruit powder mixed with saline		Rats	↑Pan T cells, CD4^+^/CD8^+^ T cells	(Srikumar et al., 2007)
			Noise-induced stress (extract = 1 g/kg/day for 48 days, p.o.; negative control: Normal saline)	↑IL-2, IFN-gamma	
				↓IL-4	
Triphala	Aqueous fruit powder suspension in 2%gum acacia	PHA induced T-lymphocyte (human blood isolation) proliferation (extract 50, 100 ug/ml for 4 days)	Mice	↓T-lymphocyte proliferation (MAC = 50 ug/ml)	(Sabina et al., 2009)
			CFA-induced arthritis (extract = 500 and 1,000 mg/kg, p.o. for 5 days)	↓Complement activity (MAC = 500 mg/ml)	
			Intraperitoneal SRBC-induced humoral antibody titer (extract = 500 and 1,000 mg/kg, p.o. for 7 days)	↓Antibody titer response (MAC = 500 mg/ml)	
			DTH: SRBC-induced foot paw swelling (extract = 500 and 1,000 mg/kg, p.o. for 4 days)	↓DTH response (MAC = 500 mg/ml)	
Triphala	95% ethanol		healthy volunteer (extract = 1,050 mg/day, p.o. for 2 weeks)	↑Cytotoxic T-cell (CD3^−^CD8^+^CD45^+^) and natural killer cells (CD16^+^CD56^+^CD45^+^)	(Phetkate et al., 2012)
*P. emblica* L	90% ethanol	Lymphocyte isolation from cromium (10 μg/ml)-induced immunosuppressed rats (extract = 100 μg/ml)		↓Cytotoxicity of chromium (MAC = 100 μg/ml)	(Sai Ram et al., 2002)
				↓Lymphocyte suppresion	
				↑Restore IL-2, IFN-gamma production	
				↓Apoptosis and DNA fragmentation	
*P. emblica* L	95% ethanol		Mice	↓CD8^+^ T cell, ↑CD4^+^(Th)	(Singh et al., 2015)
			Arsenic induced immunotoxicity (extract = 500 mg/kg, p.o. for 30 days; negative control: 2% gum acacia solution in distilled water)	↑T (CD3^+^) and B(CD19^+^) sub cells population	
*T. chebula* retz	Ethanol		Rats	↑Phagocytic activity	(Aher et al., 2010)
			SRBC-induced immune response (extract = 100 mg/kg, p.o. for 11 days; negative control: 1% gum acacia solution in distilled water, cyclophosphamide 100 mg/kg; positive control: SRBC-sensitized rats)	↑Neutrophil, lymphocyte, and monocyte	
				↑Phagocytic activity	
				↑Serum immunoglobulin	
*T. chebula* retz	70% ethanol		Rats	↑Liver mitochondrial enzyme (CAT, SOD, GSH)	(Aher and Wahi, 2011)
			SRBC-induced immune response (extract = 100 mg/kg, p.o. for 14 days; negative control: 1% gum acacia solution in distilled water, cyclophosphamide 100 mg/kg, positive control: SRBC- sensitized rats)	↓Liver LPO	
				↑Lymphocyte proliferation	
				↑IL-2, IL-10 and TNF-α mRNA expression in spleen cells	
*T. chebula* retz	Water		Mice	↓Ear swelling (thickness)	(Nam et al., 2011)
			DNFB-induced atopic symptoms (extract = 100 ug/ml, topical application for 8 days)	↓Eosinophil level	
				↓Atopic biomarkers (MMP-9, IL-31, and T-bet)	
*T. chebula* retz	Water		Mice	↑Antibody titer of gRBC (MAC = 200 mg/kg)	(Rubab and Ali, 2016)
			Goat RBC or ovalbumin-induced immune system (extract = 100, 200, 300, and 400 mg/kg, p.o. for 10 days)	↑IFN-gamma (MAC = 300 mg/kg)	
				↓IL-4 (MAC = 300 mg/kg)	
				↑Lymphocye proliferation and macrophage response	
				↑Bone marrow cellularity and WBC count (MAC = 300 mg/kg)	
*T. chebula* retz	Methanol extract followed by water fraction	Compound 48/80-induced histamine release from RPMC (extract = 0.005–1.0 g/kg)	Compound 48/80-induced anaphylactic shock in mice (extract = 0.001–1.0 g/kg, i.p.; negative control: Saline)	↓Histamine release (MAC = 0.01 g/kg)	(Shin et al., 2001)
			Anti-DNP IgE-indcued passive cutaneous anaphylaxis reaction in rats (extract = 0.001–1.0 g/kg, p.o.)	↓Mortality rate (MAC = 0.01 g/kg)	
				↓Serum histamine level (MAC = 0.1 g/kg)	
				↓Passive cutaneous anaphylaxis reaction (MAC = 1.0 g/kg)	
				↑IgE-mediated TNF-α production (MAC = 1 mg/ml)	

NBT, nitro blue tetrazolium; PHA, phytohemagglutinin; RPMC, rat peritoneal mast cells.

In theory, it is accepted that a balance of the numbers and ratios of Th cells and Tc cells is considered to be a homeostasis marker of the intrinsic immune system. Significant increments in Th cells, Tc cells, and natural killer cells following exposure to Triphala and its active constituents could confirm an additional improvement in the general cellular-mediated immunity of these agents in order to deal with harsh environments and exogenous microorganisms.

Although intranasal corticosteroids are now considered to be the most effective treatment for AR, their mechanism of action acts mainly preferentially through immunosuppression rather than immunomodulation (Bousquet et al., 2008; Hossenbaccus et al., 2020). It will be of interest to find any alternative treatments having dual immunostimulatory and immunosuppressive effects. Luckily, Triphala and its active constituents possess these properties. Thus, this polyherbal formulation might be an important potential candidate in the area of AR therapeutic development.

### Triphala: Respiratory System

Following exposure to allergens or the start of the pollen season, some AR patients may have symptoms of cough (dry cough or a cough containing phlegm that is loose in character) together with nasal symptoms (Galway and Shields, 2019). Chronic cough and bronchoconstriction may be caused by bronchial hyper-responsiveness (BHR), which has been reported in AR patients even without asthma (Eggleston, 1988; Polosa et al., 2000). The relationship between AR and BHR may be explained by the following: 1) evidence of the neuronal connection between the upper and lower airways in which the exposure of nasal mucosal to certain stimuli, such as allergens and histamine, has been found to be associated with bronchospasm, and 2) direct contact with local topical nasal inflammatory or systemic inflammatory circulating cytokines from the upper airways might cause inflammation of the lower airways (Braunstahl, 2005; Taylor-Clark et al., 2005; Undem and Taylor-Clark, 2014). IL-13, in particular, which has been reported to not only mediate airway inflammation but is also considered to be a compounding factor driving BHR, may play a role in both upper and lower airway communication (Townley et al., 2009).

The effects of Triphala and its constituents on the lower respiratory system have been reported mainly in animal models. *P. emblica* L. and *T. chebula* Retz. extracts have the ability to alleviate coughs induced by mechanical stimulation and citric acid, respectively (Table 6) (Nosál’Ováx et al., 2003; Nosalova et al., 2013). *T. bellirica* (Gaertn.) Roxb. itself can dose dependently reduce BHR induced by carbachol (Gilani et al., 2008). One study showed that Triphala alleviated bronchial hyper-reactivity via immunomodulation and anti-oxidative pathways in OVA-induced asthma in murines (Horani et al., 2012). Interestingly, some of the airway hypersensitivity endpoints using a whole-body plethysmograph, such as improvement of the pulmonary Penh value, have revealed that Triphala is superior to classical treatments such as the corticosteroid budesonide (Horani et al., 2012). However, although the immunomodulatory effects of these two treatments seem to play an important role in relieving bronchial hyper-reactivity, there are some differences in the immune response. Budesonide acts mainly through an anti-humoral mechanism by inhibiting antibody production. On the other hand, Triphala has no effect on antibody production, but its effects are selective toward lymphocyte distribution in both intra- and extrapulmonary organs (Horani et al., 2012).

**TABLE 6:** Triphala and its constituents in the respiratory system (Antitussive effect + Bronchodilator).

Herbs	Extracts	Models		Results	References
		*In vitro*	*In vivo*/*Ex-vivo*		
*P. emblica* L	Absolute ethanol		Cats	↓number of cough efforts, cough frequency, and intensity during expiration and inspiration (MAC = 50 mg/kg)	(Nosál’Ováx et al., 2003)
			Mechanical stimulation-induced coughing (extract = 50,200 mg/kg, p.o.; positive control: Codeine 10 mg/kg, i.p., dropropizine 100 mg/kg, i.p.)		
*T. chebula* retz	Polysaccharide extract (water-extracted carbohydrate polymer)		Guineapig	↓number of cough efforts	(Nosalova et al., 2013)
			Aerosol citric acid (0.3 M) induced cough (extract = 50 mg/kg p.o.; negative control: Water; positive control: Codeine phosphate 10 mg/kg)		
*T. bellirica* (gaertn.) roxb	70% methanol		Rats	↓bronchoconstriction (18, 39, and 98% inhibition at dose of 30, 100, and 300 mg/kg, respectively)	(Gilani et al., 2008)
			Carbachol (1 μM/kg)-induced bronchoconstriction (extract = 30,100, and 300 mg/kg, i.v.; positive control: salbutamol = 0.001, 0.03, and 0.1 mg/kg, i.v.)		
			Carbachol; CCh 1 μM/kg and K^+^ 80 mM/kg induced-isolated guineapig’s trachea (extract = 0.1–10 mg/ml; negative control: Saline; positive control: Dicyclomine, nifedipine, atropine)	↓tracheal contraction (IC_50_ of CCh = 3.3 mg/ml and IC_50_ of K^+^ = 8.5 mg/ml)	

## Perspectives and Conclusion

AR inflammation is a complex disease that involves several different types of cells and mediators. Specific treatments that aim to manipulate only a single mediator or one type of cell are less likely to be effective. On this basis, it is necessary to employ agents with a broader spectrum of action that can alleviate the complex symptoms of this disease. The realm of complementary and alternative medicine has gained greater attention since much evidence from different study models for specific pharmacological properties (as well as the results from clinical trials) has been accumulated, and that increases the reliability and confidence of using this lineage of medicine. Recently, several plants and their constituents are approved to be safe, effective, and less expensive for managing the development and progression of various diseases, and many of them are already on the WHO’s list of essential medicines. Therefore, Triphala which contains three different plants may be one of the effective remedies to alleviate AR-related symptoms. Experimental data from previous *in vitro* and *in vivo* studies of Triphala or individual plants in this remedy have demonstrated potential natural compounds that may be the main acting molecules able to promote the attenuation of AR symptoms. The current review (as shown in Table 2) helps reveal phytochemicals in Triphala that exhibit the promising pharmacological activities that may provide potential benefits in alleviating AR symptoms. Specifically, Triphala contains flavonoids like quercetin. The benefits of quercetin on AR may be through its ability to decrease histamine release from mast cells, and that may help reduce the process of allergic inflammation at the early phase. Moreover, its effects on suppressing the production of cytokines and eosinophil chemoattractants may help reduce the late phase inflammation and eventually the AR symptoms. However, in the clinical view, it may be challenging to utilize quercetin as a monotherapy since the pathophysiology of AR is complex. It would be more convincing to incorporate this compound in the standard regimen if there is more scientific data identifying its pharmacological targets that explain the specific effects of quercetin on alleviating AR. Besides quercetin, kaempferol is another flavonoid that shows interesting inhibitory effects on inflammation in the airways. This compound can suppress mast cell stimulation, production, and release of inflammatory cytokines and chemokines. Triphala also contains ellagic acid which has been shown to reduce histamine release and inflammation in the lung via suppressing the activity of NF-κB, and these effects help speed up the resolution of allergic airway inflammation. Another functional compound found in Triphala is gallic acid which has been reported to suppress nasal histamine release and inflammation. This compound is one of the active components that may help reduce nasal symptoms caused by autacoids and inflammatory cytokines. Similarly, chebulagic acid has been shown to possess anti-inflammatory properties by reducing cytokine production and inflammatory enzymes including COX-2 and LOX. Moreover, Triphala contains various natural compounds that may help suppress allergic responses through their anti-oxidant potential. Those compounds include vitamin C, chebulinic acid, and emblicanin. These compounds function mainly in scavenging free radicals and reducing oxidative stress; therefore, they may be the responsible compounds to help suppress exacerbation of AR-related symptoms. In short, Triphala is explored to be an important source of bioactive compounds able to be used for AR treatments. The orchestration of these compounds may create synergistic effects to decrease the exacerbation of inflammatory response and oxidative stress that lead to AR development.

The anti-inflammatory, immunomodulatory, antioxidant, and antibacterial activities of Triphala are similar to those of other herbal formulas clinically proven to treat AR effectively, such as Shi-Bi-Lin (a Chinese herbal formula), butterbur, and *Nigella sativa* (black caraway or black cumin). However, there have not been any *in vivo* studies that utilize a direct model for AR to test the effects of Triphala. The ovalbumin-induced guinea pig model of AR could well be a proper assay to definitively evaluate if Triphala can reduce AR-associated inflammation. However, there is a limitation to these types of study models because it is not a direct AR model to give us enough reliable information specific to AR. It would provide strong evidence if in the future there are direct experimental designs specifically developed for animal AR models to verify the effectiveness of Triphala.

During allergic inflammation, the nasal epithelial barriers of AR patients appear to become impaired. Consequently, this condition leads to the development of sinusitis and otitis media, with effusion caused by bacteria, viruses, and fungi. Although Triphala shows promise in being an effective bacteriostatic/bactericidal agent, most studies have focused only on oral streptococci. Thus, further studies are needed to address whether Triphala is effective in killing other types of bacteria, as well as viruses and fungi that are related to allergic rhinosinusitis. Additionally, clinical studies should be conducted to verify the efficacy of this herbal formula. As a step toward the clinical investigation of Triphala for its efficacy and safety, our research team has created the study design/clinical trial, and the study protocol has already been registered in Thai Clinical Trials Registry (TCTR20191008005). Moreover, study models for pharmacokinetics are crucial to fulfill all aspects of the pharmacology of Triphala and to obtain valuable information about the active compound profiles. If the accumulated data clearly show that Triphala can be used effectively to inhibit inflammation, modulate immunity, alleviate unwanted symptoms in patients with AR, and prevent AR-related infections, this will provide a novel alternative treatment for patients. For example, Triphala could be used as a monotherapy in patients with mild AR. For patients with moderate-to-severe AR (for which intranasal corticosteroids are usually prescribed) but who do not want to use steroidal drugs, Triphala may be used in combination with an antihistamine. For patients using steroids who have inadequate control of AR, Triphala may be added to the treatment plan to reduce the use of high-dose steroids. Similarly, for patients who require immunotherapy, a Triphala supplement may offer a faster and better response to immunotherapy.

In conclusion, Triphala exhibits beneficial effects if used as an alternative treatment for AR. However, extensive investigations in both animals and humans are first needed to be certain about its definitive mechanisms of action and the efficacy/safety of treatments. Although most studies reported the pharmacological effects of Triphala in a reasonable range of concentrations, some studies evaluated the effects of Triphala at relatively high doses. This may create concerns about its low efficacy and toxicity. With this, specifying the efficacy and adverse effects of Triphala is a vital base to ensure its safe use. Triphala should be carefully examined further to gain adequate data about toxic target organs, safe dose range, safety window of effective dose, and minimum toxic dose. Considering the actual applications, appropriate dosage and course of treatment are required to be precisely adjusted to ensure that Triphala is safe and effective. The accumulated evidence obtained from such studies will be beneficial to properly prescribe Triphala to individual AR patients with different clinical symptoms and varying degrees of severity in order to achieve the highest efficacy with the fewest side effects.
