# Supplementary material for: Pharmacological Benefits of Triphala: A Perspective for Allergic Rhinitis
Source: Front Pharmacol. 2021 Apr 30;12:628198. doi: 10.3389/fphar.2021.628198 (PMC8120106; doi:10.3389/fphar.2021.628198)
Supplement: Supplementary file 1 [file DataSheet1.PDF]

Supplementary table 1: Article quality assessment

Articles	Developing the concept									Concept and methods										Reporting data and outcomes									Conclusion
							In vivo					Experimental design																	
	Relevent of triphala (review literature of potential used)	Novelty of study	Main active compounds	Plant sustainability	Ethical concerns before starting project	Specific methods and tools	Specific methods leads to therapeutics relevant	Translation of traditional medicine	Triphala compounds been registered	Define botanical material	Phytochemical profile	Animal species/ cell types	Group sized	Extract disolved	Define positive and negative controls	Dose range	Toxicity doses	Statistical evaluation	Appropriate methadologies	Resulting pharmacological data potentially linked to common (ubiquitous) compounds with known effects	suitable statistics (N $\geq$3)	Specific results (cell types and signaling pathway)	Use of commercially available kits	Cytotoxicity test (non-toxic dose)	Methods of antioxidant tested	Proof of hypothesis	Highlight limitation	Clinical implicaiton	Specific future perspective
Naik et al., 2006	+	+	+	+	n/a	+	n/a	-	+	+	+	n/a	-	+	+	+	-	-	+	+	-	-	-	-	+	+	-	+	-
Babu et al., 2013	+	+	+	+	n/a	+	n/a	-	+	+	+	n/a	+	+	+	+	-	+	+	+	+	-	-	-	+	+	-	+	+
Gajendra et al., 2016	+	+	+	+	n/a	+	n/a	-	+	-	+	n/a	-	+	+	+	-	-	+	+	-	-	-	-	+	+	-	+	+
Varma et al., 2016	+	+	+	+	n/a	+	n/a	-	+	+	+	+	+	+	+	+	+	+	+	+	+	+	+	+	+	+	-	+	+
Takauji et al., 2016	+	+	+	+	n/a	+	n/a	-	+	-	-	+	+	+	-	-	-	+	+	+	+	-	-	-	+	+	-	+	+
Sharma et al. 2011	+	+	-	+	-	+	+	-	+	-	-	+	+	n/a	+	-	-	+	+	+	+	-	-	-	+	+	-	+	-
Srikumar et al., 2006	+	+	-	+	+	+	+	-	+	-	-	+	+	+	+	-	-	+	+	+	+	-	+	-	+	+	-	+	-
Hazra et al., 2010	+	+	+	+	+	+	+	-	+	+	+	+	+	+	+	+	-	+	+	+	+	-	-	-	+	+	-	+	+
Liu et al., 2008	+	+	+	+	n/a	+	n/a	-	+	-	+	n/a	n/a	+	+	+	-	+	+	+	+	-	-	-	+	+	-	+	+
Charoenteeraboon et al., 2010	+	+	+	+	n/a	+	n/a	-	+	+	+	+	+	+	+	+	-	+	+	+	+	-	-	-	+	+	-	+	-
Mehrotra et al., 2011	+	+	+	+	n/a	+	n/a	-	+	-	+	n/a	n/a	+	-	+	-	-	+	+	-	-	-	-	+	+	-	+	-
Mahesh et al., 2009	+	+	-	+	+	+	+	-	+	-	-	+	+	+	+	-	-	+	+	+	+	-	-	-	+	+	-	+	-
Rasool and Sabina, 2007	+	+	+	+	+	+	+	+	+	+	+	+	+	+	+	+	-	+	+	+	+	-	-	-	-	+	+	+	+
Prabu et al., 2008	+	+	+	+	+	+	+	-	+	-	+	+	+	+	+	+	-	+	+	+	+	-	-	-	-	+	-	+	+
Sireeratawong et al., 2013	+	+	-	+	+	+	+	+	+	+	-	+	+	+	+	+	-	+	+	+	+	-	-	-	-	+	-	+	-
Kalaiselvan and Rasool, 2015	+	+	+	+	+	+	+	+	+	+	+	+	+	+	+	-	-	+	+	+	+	+	+	-	+	+	-	+	+
Kalaiselvan and Rasool, 2016	+	+	+	+	+	+	+	+	+	+	+	+	+	+	+	+	+	+	+	+	+	+	+	+	-	+	-	+	-
Shanmuganathan and Angayarkanni, 2018	+	+	+	+	+	+	n/a	-	+	-	+	+	n/a	+	-	+	-	+	+	+	+	+	+	+	-	+	-	+	+
Deshmukh et al., 2010	+	+	-	+	+	+	+	+	+	-	-	+	+	+	+	+	-	+	+	+	+	-	-	-	-	+	-	+	-
Golechha et al., 2011	+	+	+	+	+	+	+	+	+	+	+	+	+	+	+	+	-	+	+	+	+	-	+	-	+	+	-	+	-
Muthuraman et al., 2011	+	+	+	+	+	+	+	-	+	+	+	+	+	+	+	+	-	+	+	+	+	-	-	-	+	+	-	+	+
Pradyumna Rao et al., 2013	+	+	+	+	+	+	+	-	+	-	+	+	+	+	+	+	-	+	+	+	+	+	+	+	-	+	-	+	+
Golechha et al., 2014	+	+	+	+	+	+	+	-	+	+	+	+	+	+	+	+	-	+	+	+	+	-	-	-	+	+	-	+	+
Middha et al., 2015	+	+	+	+	+	+	+	-	+	+	+	+	+	+	+	+	+	+	+	+	+	+	+	+	+	+	-	+	+
Nair et al., 2010	+	+	+	+	+	+	+	-	+	+	+	+	+	+	+	+	+	+	+	+	+	+	+	n/a	n/a	+	-	+	-
Nair et al., 2012	+	+	+	+	+	+	+	-	+	+	+	+	+	+	+	+	+	+	+	+	+	+	+	-	n/a	+	-	+	-
Sukakul et al., 2013	+	+	-	+	-	+	+	-	+	-	-	+	+	+	-	-	-	+	+	-	+	-	-	-	n/a	+	-	+	+
Bag et al., 2013	+	+	+	+	+	+	+	-	+	+	+	+	+	+	+	+	-	+	+	+	+	-	-	-	+	+	-	+	+
Ibne Jami et al., 2014	+	+	+	+	+	+	+	-	+	+	+	+	+	+	+	+	+	+	+	+	+	-	-	+	n/a	+	+	+	+
Yang et al., 2014	+	+	+	+	n/a	+	n/a	-	+	+	+	+	n/a	+	+	+	+	+	+	+	+	+	+	+	n/a	+	-	+	-
Sireeratawong et al., 2014	+	+	+	+	+	+	+	-	+	+	+	+	+	+	+	+	-	+	+	+	+	-	-	-	n/a	+	-	+	-
Kirubanandan et al., 2015	+	+	+	+	+	+	+	-	+	+	+	+		+	+	+	-	+	+		+	-	-	-	n/a	+	+	+	+
An et al., 2016	+	+	+	+	+	+	+	+	+	+	-	+	+	+	+	+	+	+	+	+	+	+	+	+	+	+	-	+	+
Murdock, 2015	+	+	-	-	+	+	+	+	+	-	-	+	+	+	+	-	-	+	+	+	+	-	-	-	-	+	-	+	+
Rahimi et al., 2018	+	+	+	+	n/a	+	n/a	-	+	+	+	+	n/a	+	-	+	-	+	+	+	+	+	+	+	n/a	+	-	+	+

Jayesh et al., 2018	+	+	-	+	n/a	+	n/a	-	+	+	-	+	n/a	+	+	+	+	+	+	+	+	+	+	+	+	+	-	+	-
Tanaka et al., 2018	+	+	+	+	n/a	+	n/a	-	+	+	+	+	n/a	+	-	+	+	+	+	+	+	+	+	+	+	+	-	+	+
Chauhan et al., 2018	+	+	+	+	+	+	+	-	+	+	+	+	+	+	+	+	+	+	+	+	+	-	-	+	n/a	+	-	+	+
Srikumar et al., 2005	+	+	-	+	+	+	+	-	+	-	-	+	+	-	+	+	-	+	+	-	+	-	-	-	-	+	-	+	-
Srikumar et al., 2007	+	+	-	+	+	+	+	-	+	-	-	+	+	-	+	+	-	+	+	-	+	+	+	-	-	+	-	+	+
Sabina et al., 2009	+	+	-	+	+	+	+	-	+	+	-	+	+	+	+	+	-	+	+	+	+	-	-	+	n/a	+	+	+	+
Sai Ram et al., 2002	+	+	-	+	n/a	+	n/a	-	+	-	-	+	n/a	+	-	+	+	+	+	+	+	-	+	+	+	+	-	+	-
Singh et al., 2015a	+	+	-	+	+	+	+	-	+	-	-	+	+	+	+	+	-	+	+	+	+	-	+	+	n/a	+	-	+	+
Aher et al., 2010	+	+	-	+	+	+	+	-	+	-	-	+	+	+	+	-	-	+	+	-	+	-	-	-	n/a	+	+	+	+
Aher and Wahi, 2011	+	+	-	+	+	+	+	+	+	+	-	+	+	+	+	-	-	+	+	+	+	+	+	-	+	+	-	+	+
Nam et al., 2011	+	+	-	+	+	+	+	+	+	+	-	+	+	+	+	-	-	+	+	+	+	+	+	-	n/a	+	+	+	-
Rubab and Ali, 2016	+	+	+	+	+	+	+	+	+	+	+	+	+	+	+	+	-	+	+	+	+	+	+	-	n/a	+	-	+	-
Shin et al., 2001	+	+	-	+	+	+	+	+	+	+	-	+	+	+	+	+	+	+	+	+	+	+	+	+	n/a	+	-	+	+
Nosál'Ováx et al., 2003	+	+	-	+	+	+	+	+	+	-	-	+	+	+	+	+	-	+	+	+	+	-	-	-	-	+	-	+	-
Nosalova et al., 2013	+	+	+	+	+	+	+	+	+	+	+	+	+	+	+	-	-	+	+	+	+	+	-	-	n/a	+	-	+	-
Gilani et al., 2008	+	+	-	+	+	+	+	+	+	-	-	+	+	+	+	+	+	+	+	+	+	+	-	-	n/a	+	-	+	-

Clinical study quality assessment [the Good Research for Comparative Effectiveness (GRACE) tool]

	Adequate treatment	Adequate outcome	Objectec outcomes	Valid outcomes	Similar outcomes	Covariates recorded	New initiators	Concurrent comparator	Covariates accounted for	Immortal time bias	Sensitivity analysis
Author (year)	D1	D2	D3	D4	D5	D6	M1	M2	M3	M4	M5
Phetkate et al., 2012	+	+	+	+	+	-	+	+	+	+	+
